# Detailed Seed Cone Morpho-Anatomy Provides New Insights into Seed Cone Origin and Evolution of Podocarpaceae; Podocarpoid and Dacrydioid Clades

**DOI:** 10.3390/plants12223903

**Published:** 2023-11-19

**Authors:** Raees Khan, Robert S. Hill, Veit M. Dörken, Ed Biffin

**Affiliations:** 1 School of Biological Sciences, The University of Adelaide, Adelaide, SA 5005, Australia; bob.hill@adelaide.edu.au; 2 CAS Key Laboratory for Plant Diversity and Biogeography of East Asia, Kunming Institute of Botany, Chinese Academy of Sciences, Kunming 650201, China; 3 State Herbarium of South Australia, Adelaide, SA 5005, Australia; edward.biffin@adelaide.edu.au; 4 Department of Biology, University of Konstanz, 78457 Konstanz, Germany; veit.doerken@uni-konstanz.de

**Keywords:** conifers, developmental biology, dispersal, evolution, fossils, leaf dimorphism, paleobotany

## Abstract

The study of reproductive morphology and trait evolution provides a vital insight to understand the evolutionary history of plants. The conifer family Podocarpaceae has a remarkable diversity of seed cones, with distinct morphology among the genera and with conifers in general. However, we lack a good understanding of the seed cone morpho-anatomy and trait evolution of Podocarpaceae. We investigated detailed seed cone morpho-anatomy using staining and sectioning techniques to clarify the anatomical, morphological diversity and evolution of functional traits. The presence of a fleshy receptaculum is a characteristic feature of both clades. However, species of *Retrophyllum, Afrocarpus* and some species of *Nageia* and *Podocarpus* form a fleshy sarcotesta-like seed coat, lacking a fleshy receptaculum. The ancestral state reconstructions show a shift between and sometimes within the genus. Although both clades demonstrate fleshiness as an ancestral trait, the shift in fleshy structures provides evidence for complex multiple evolutions of fleshy morphologies. These seed cone traits (e.g., fleshiness and size), along with the broad, flattened and well-adapted (leaf dimorphism) foliage in both clades, are largely congruent with efficient light harvesting and bird dispersal. These traits make these two clades well adapted to their environment, when growing in communities including tall and broad-leaved angiosperms (closed-canopy angiosperm forests), compared to other podocarps, making them more successful in achieving a wider distribution and species richness.

## 1. Introduction

Podocarps are unique among conifers in their ability to successfully compete with angiosperms, especially in tropical forests [1]. Podocarps achieved this capacity by evolving some functionally significant traits, e.g., flattened broad leaves, fleshy seed cones and large seed sizes [2,3,4,5]. Podocarps have an amazing diversity of fleshy seed cones and have evolved several complex functional structures in different genera [6,7,8,9,10]. Recent phylogenetic analyses show three major clades in the Podocarpaceae, i.e., Prumnopityoid, Podocarpoid and Dacrydioid, along with some separate and distinct lineages [4,11]. The Podocarpoid and Dacrydioid clades have evolved an amazing diversity in both leaf and seed traits [4,12,13,14]. The Podocarpoid clade has a suggested crown age of approximately 75 Ma (54–85 Ma) and is the largest genus within Podocarpaceae (consisting of more than 130 species), distributed in Southeast Asia and through much of the Southern Hemisphere [13,15]. The Dacrydioid clade is the second most species-rich clade and includes three genera (*Dacrydium*, *Dacrycarpus* and *Falcatifolium*) with a suggested crown age of approximately 75 Ma (54–95 Ma) and is predominantly distributed in the Southern Hemisphere [4,13]. Some studies have evaluated seed cone evolution in conifers based on morpho-anatomy, and some have used model-based ancestral reconstruction [6,8,14,16,17,18,19]. However, detailed and comprehensive studies of the seed cone morpho-anatomy are still lacking. Klaus and Matzke [6], in concluding that the ancestral state for the Podocarpaceae seed cone is fleshy, considered the Podocarpoid clade genera *Afrocarpus* and *Retrophyllum*, and some species of *Nageia* and *Podocarpus* as non-fleshy. The study by Chen et al. [19] considers the epimatium of *Dacrydium*, *Falcatifolium*, *Microcachrys* and *Lepidothamnus* as non-fleshy and reported a complete absence of fleshy receptacles in *Nageia* in their ancestral reconstructions. Although some studies have been undertaken to evaluate different aspects of the reproductive cycles of *Acmopyle*, *Podocarpus*, *Dacrydium* and *Dacrycarpus* [14,16,17,18], no comprehensive studies are available on detailed seed cone morpho-anatomy and the evolution of functional traits and structures for both the Podocarpoid and Dacrydioid clades. Similarly, because seed cone morphology is complex in podocarps and there has been a lack of detailed studies on the seed cone morpho-anatomy, it is difficult to correlate fossil seed cones with extant taxa [4]. The recent studies on the detailed seed cone morpho-anatomy of three paleoendemic genera [9] and the Prumnopityoid clade [10] provide vital insights into the origin and evolution of seed cone types in Podocarpaceae [20]. The goal of this study is to understand and describe the detailed morpho-anatomical structures of the two largest species rich clades, i.e., Podocarpoid and Dacrydioid clades’ seed cones, and discuss the complex evolutionary history of fleshiness in Podocarpaceae. The morpho-anatomical similarities and differences among the genera are evaluated and compared with the available related fossil data. The origin, evolution, and potential drivers of fleshy seed cone evolution among the genera of these clades are also discussed.

## 2. Material and Methods

### 2.1. Seed Cone Collection

Seed cones at different developmental stages were collected from the Australian National Botanic Gardens, Canberra, ACT, Australia; Mount Lofty Botanical Garden, South Australia; and The Tasmanian Arboretum, Devonport, Tasmania. Seed cones of *Nageia* (AN-395) and *Retrophyllum* (RB-23) were collected from preserved specimens in the State Herbarium of South Australia and the Department of Ecology and Evolution, University of Adelaide. The spirit collection of the seed cones, with voucher numbers from UOA-10 to UOA-60, are stored at the Department of Ecology and Evolution, University of Adelaide, Australia. We included *Acmopyle* in this study as an outgroup because it has a fleshy receptacle, a papery epimatium fused with the outer testa and a woody sclerotesta (endotesta). Data on seed dispersal were collected from the available literature.

### 2.2. Morphology and Distribution of the Investigated Taxa

Members of the Podocarpoid clade are usually large trees or sometimes shrubs, with broad and flattened leaves and fleshy seed cones. Representative species from each genus that cover the variation in the seed cone morphology were included here. In this study, we examined:

1. The South African *Afrocarpus falcatus* (Thunb.) C.N. Page, which forms large trees [21].
2. Two species of *Nageia*, *N. nagi* (Thunb.) Kuntze, which is an evergreen tall tree, distributed in China (introduced), Japan, Taiwan (introduced) and Vietnam [21,22] and *N. wallichiana* (Presl.) Kuntze, which is a tall tree distributed in Brunei Darussalam, Cambodia, China, India, Indonesia, Laos, Malaysia, Myanmar, Papua New Guinea, the Philippines, Thailand and Vietnam [21,22]).
3. Three species of *Podocarpus* subgenus *Podocarpus* (*P. henkelii* Stapf., *P. elongatus* (Aiton) L’Herit. Ex Persoon and *P. oleifolius* D. Don). *Podocarpus henkelii* and *P. elongatus* occur in southern Africa, while *P. oleifolius* is found in Central America [23].
4. Two species of *Podocarpus* subgenus *Foliolatus* (*P. spinulosus* (Smith) R. Br. Ex Mirbel and *P. elatus* R. Br. ex Endlicher). *Podocarpus spinulosus* and *P. elatus* are dioecious species from northeastern Australia [24].
5. *Retrophyllum comptonii* (Buchholz) C.N. Page, which is a tree endemic to New Caledonia [25].

Additionally, we examined the following members from the Dacrydioid clade:

1. *Dacrycarpus dacrydioides* (A. Richard.) de Laub., a tall tree endemic to New Zealand.
2. *Dacrydium cupressinum* Solander ex G. Forst., a tall tree endemic to New Zealand.
3. *Falcatifolium papuanum* de Laub., a tall tree endemic to Papua New Guinea [26].

Finally, we included *Acmopyle pancheri* (Brongniart et Grisebach) Pilger, which is endemic to New Caledonia [21,22,27] as an outgroup to the two main clades.

### 2.3. Taxon Processing and Sectioning 

Specimens were fixed in 200 mL of FAA (100 mL 95% ethanol + 80 mL dH_2_O + 20 mL 37% formaldehyde solution) immediately after collection. Whole seed cones, plus longitudinal and cross sections of the seed cones were photographed with a Nikon-SMZ25 stereomicroscope. For histology, seed cones were fixed for 48–72 h. The seed cones were processed for a 48 h cycle on a Sakura Tissue-Tek VIP6 Vacuum Infiltration Tissue Processor (Torrance, CA, USA). The seed cones were embedded in paraffin wax (Sakura Tissue Tek embedding centre) and then longitudinal and cross sections of 8–10 µm thickness, were produced using a Leica RM 2235 Rotary Microtome, (Wetzlar, Germany) and stained with H & E (DAKO Cover Stainer (Agilent, Santa Clara, CA, USA)) and Toluidine blue stains. The sections were brought to water and to slide. Each slide was individually stained for 10 s in Toluidine blue stain by pipetting a few drops of Toluidine blue stain into each slide. The slides were observed under light microscopy and photographed at several magnifications.

### 2.4. Measurements and Trait Reconstructions

Different morpho-anatomical and embryological characters were recorded (Table 1). The anatomical layers were measured from mature seed cones (ten seed cone replicates for each species). The measurements were taken using ImageJ 1.8.0_112 software. Seed cone morpho-anatomical trait data were collected mainly from living and Herbarium material. Quantitative seed cone data were mainly based on Herbarium observations and information in Farjon [21]. For ancestral reconstruction, we used the dated phylogeny of Khan et al. [13], based on three chloroplasts (rbcL, matK and trnL-trnF) and three nuclear genes (NEEDLY, PHYP and ITS2). The characters were mapped for their evolution using RASP 4.2-BayesTraits [28], Mesquite 3.6 [29] with maximum likelihood (ML) and the Markov chain Monte Carlo (MCMC) reconstruction method.

## 3. Results

### 3.1. Afrocarpus Seed Cone Morpho-Anatomy

**Figure 1:**
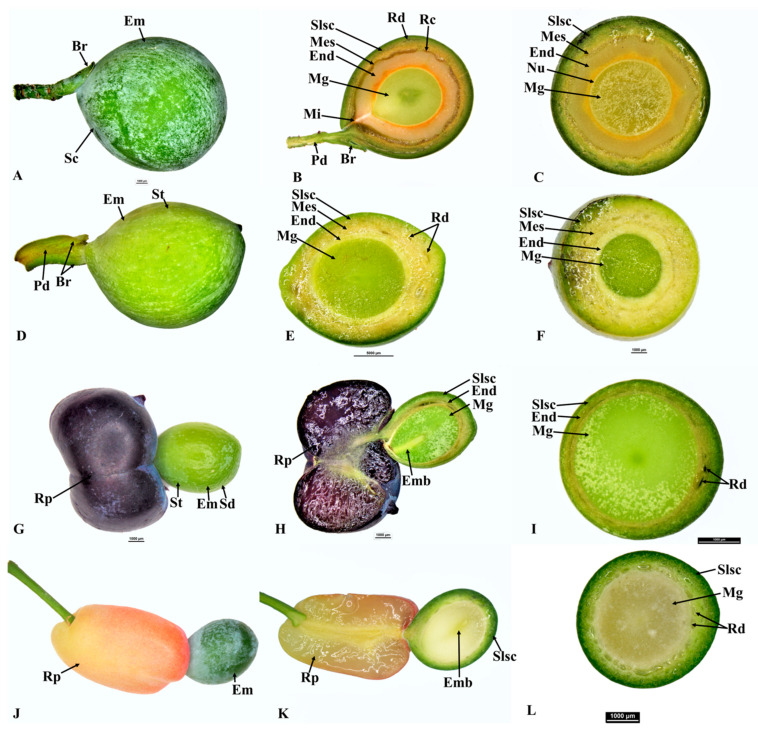
Seed cone of *Afrocarpus falcatus* (**A**), longitudinal section (**B**), cross section (**C**); *Podocarpus henkelii* (**D**), longitudinal section (**E**), cross section (**F**); *Podocarpus spinulosus* (**G**), longitudinal section (**H**), cross section (**I**); and *Podocarpus oleifolius* (**J**), longitudinal section (**K**), cross section (**L**). Shown are the epimatium (Em), bract (Br), fleshy sarcotesta-like seed coat (Slsc), mesotesta (Mes), endotesta (End), megagametophyte (Mg), micropyle (Mi), embryo (Emb), resin canal (Rc), resin duct (Rd), peduncle (Pd), receptaculum (Rp) and resin layer (Rl).

*Afrocarpus falcatus* seed cones are yellow, obovoid, and are produced abundantly annually (Figure 1A). It is wind-pollinated, and the reproductive cycle completes in one year from initiation to the formation of the mature cone. The terminal bud initiates in the form of two primordia and develops into an ovule–epimatium complex. This structure differentiates into a seed cone apex and later into an ovule with an integument and epimatium. The megaspore mother cell differentiates from other cells surrounded by the nucellus. The seed cone (12–20 × 6–14 mm) is surrounded by 1–2 bracts and the ovule is inverted. The seed cone cross sections show six major zones: (i). Epimatium: 8–14 layers of small irregular isodiametric cells. Scattered sclereids cells are also present in this zone. (ii). Exotesta: 10–18 layers of round and elongated cells. Multiple vascular traces (four ascending and several descending), as well as resin canals, were observed in this zone. Scattered sclereids cells are also present. The epimatium is connate with the exotesta forming a fleshy sarcotesta-like seed coat (Section 3.6, a–c in the figure). (iii). Mesotesta: 3–5 layers of elongated cells. The vascular bundles from the exotesta enter this zone. These layers are highly resinous. (iv). Endotesta: 20–32 compact layers of small dense cells. The endotesta is hard and stony when mature, forming a sclerified sclerotesta-like structure. (v). Nucellus: The protective cover of the embryo consisting of 4–10 layers of dense and smaller cells and (vi). Gametophyte: 10–22 layers of cells with a mature embryo which is about 0.8–1.2 × 0.3–0.5 mm. 

### 3.2. Nageia Seed Cone Morpho-Anatomy

*Nageia nagi* produces brown globose (12–18 × 10–16 mm) seed cones that are wind-pollinated. In its native range, the reproductive cycle initiates in February and completes in November to December. Pollination occurs in April–May. The ovule is inverted. The seed cone cross sections show five major zones (Supplementary Figure S1): (i). Epimatium: 4–8 layers of small and large irregular isodiametric cells. (ii). Exotesta: 12–20 layers of round and elongated cells. Several vascular bundles and resin ducts are present in this layer. The epimatium is connate with the exotesta forming a fleshy sarcotesta-like seed coat. (iii). Endotesta: 10–18 compact layers of small dense cells. The sclerotesta is hard and stony when the seed cone is mature, forming a sclerified sclerotesta-like structure. (iv). Nucellus: 6–12 layers of dense and smaller cells. (v). Gametophyte: 12–20 layers of cells with a mature embryo which is about 0.8–1.5 × 0.3–0.4 mm. The *N. nagi* seed cone has no fleshy receptacle, similar to *N*. *fleuryi*, *N*. *formosensis* and *N*. *maxima*, but distinct from *N. wallichiana*, where the seed cones have a fleshy receptaculum (Table 1).

### 3.3. Podocarpus Seed Cone Morpho-Anatomy

#### 3.3.1. *Podocarpus henkelii*

**Figure 2:**
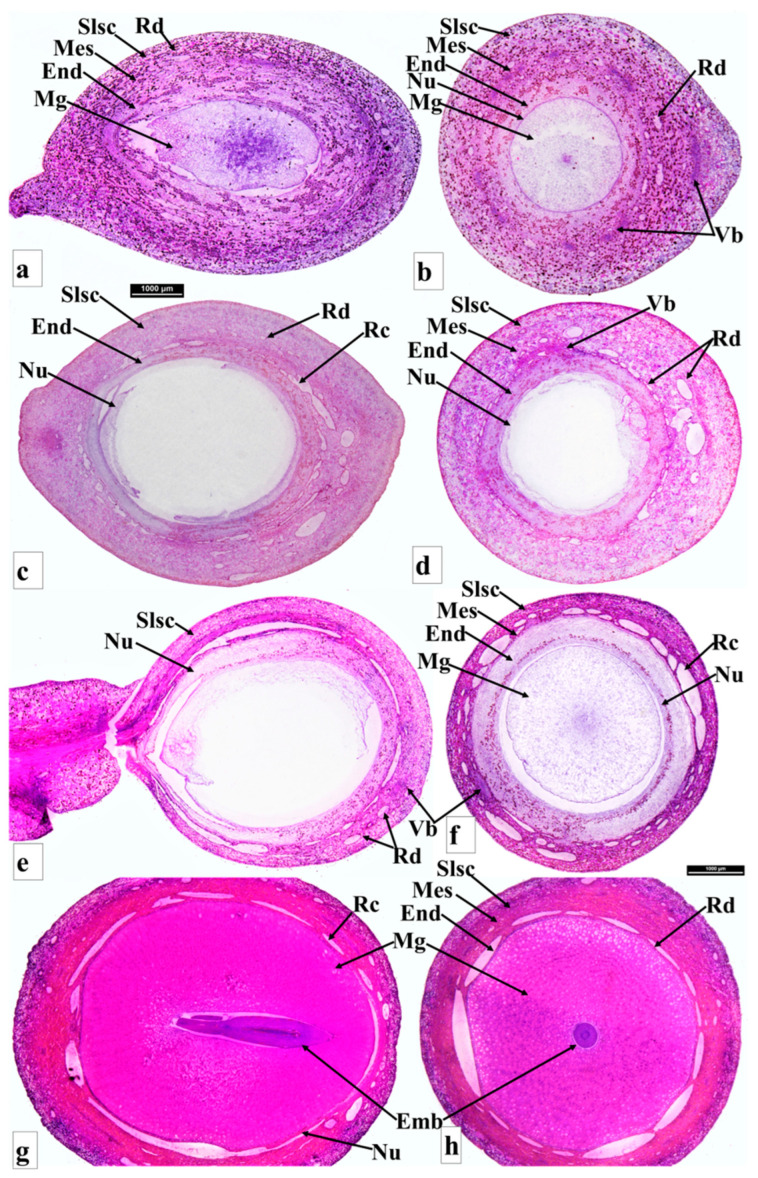
Seed anatomy of *Podocarpus oleifolius* longitudinal section (**a**), cross section (**b**); *P. henkelii* longitudinal section (**c**), cross section (**d**); *P*. *elongatus* longitudinal section (**e**), cross section (**f**); and *P*. *elatus* longitudinal section (**g**), cross section (**h**). Shown are the epimatium (Em), bract (Br), fleshy sarcotesta-like seed coat (Slsc), mesotesta (Mes), endotesta (End), megagametophyte (Mg), embryo (Emb), resin canal (Rc), resin duct (Rd) and vascular bundles (Vb).

*Podocarpus henkelii* produces obovoid seed cones with one inverted ovule and completes its reproductive cycle in one year. The seeds are yellow–green and are positioned on a non-fleshy receptacle (Figure 1D). The seed cones are 15–20 mm long and 8–12 mm wide. The seed cone cross section shows six zones: (i). Epimatium: 12–16 layers of small, irregular isodiametric cells. Sclereids are present in this zone. The epimatium is fleshy and fused with the exotesta surrounding the whole seed, forming a fleshy sarcotesta-like seed coat. (ii). Exotesta: 12–18 layers of round and elongated cells. Several vascular traces, small resin ducts and large resin canals and sclereids are present in this zone (Figure 2c,d). (iii). Mesotesta: 16–22 layers of dense round and rectangular sclerenchymatous cells. (iv). Endotesta: 12–20 compact layers of small isodiametric cells. The mature endotesta is woody and stony, forming a sclerified sclerotesta-like structure. (v). Nucellus: 6–12 layers of isodiametric cells, forming the protective cover of the embryo. (vi). Gametophyte: 14–24 layers with a straight embryo (about 0.65–1.2 × 0.2–0.4 mm in size). 

#### 3.3.2. *Podocarpus elongatus*

*Podocarpus elongatus* has an oblong seed cone with one inverted ovule positioned on the bright red receptaculum. The seed cone reproductive cycle completes in one year. The seed cone cross section shows seven major zones: (i). Epimatium: 6–10 layers of small irregular isodiametric cells. Sclereids are present in this zone. The epimatium is papery and fused with the outer testa surrounding the whole seed. (ii). Exotesta: 8–13 layers of round and elongated cells. Several enlarged vascular traces and normal-sized resin canals and sclereids are present in this zone. The epimatium is papery and fused with the exotesta surrounding the whole seed. (iii). Mesotesta: 3–5 layers of dense, round, and rectangular sclereid cells. (iv). Endotesta: 6–12 layers of large and small irregular and isodiametric cells (Figure 2e,f). The cells are sclerified when the seed cone is mature, but they are not woody. (v). Nucellus: 8–14 layers of large and small irregular and isodiametric cells, forming the protective cover of the embryo. (vi). Gametophyte: 14–18 layers of cells with a straight embryo (about 0.5–0.9 × 0.2–0.3 mm in size). (vii). Receptacle: 12–25 layers of small and large rounded cells. The longitudinal section shows that three vascular bundles enter the receptaculum. The central vascular bundle enters the seed.

#### 3.3.3. *Podocarpus oleifolius*

**Figure 3:**
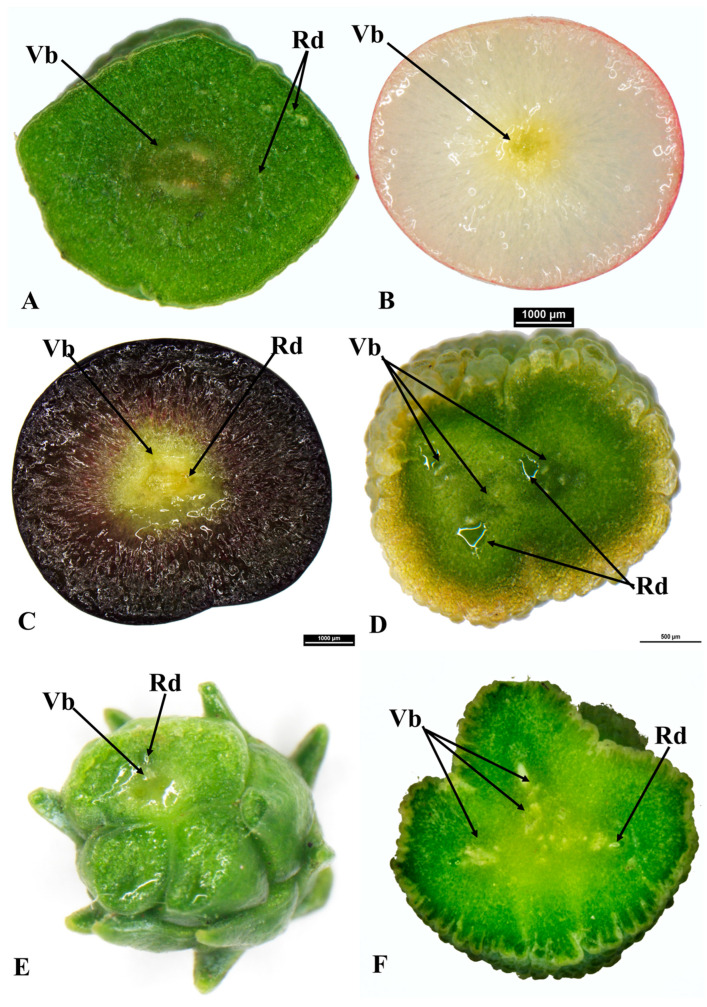
Cross sections of the receptaculum of *Podocarpus elongatus* (**A**); *Podocarpus oleifolius* (**B**); *Podocarpus spinulosus* (**C**); *Dacrycarpus dacrydioides* (**D**); *Dacrydium cupressinum* (**E**) and *Acmopyle pancheri* (**F**), showing vascular bundles (Vb) and resin ducts (Rd).

*Podocarpus oleifolius* produces elongated-oblong fleshy seed cones (12–18 × 4–7 mm) and completes its reproductive cycle in one year (Figure 1J,K). One or two inverted ovules are placed on the yellowish red fleshy receptacle with two small bracts. The seed cone cross section shows seven zones: (i). Epimatium: 8–12 layers of small and large isodiametric cells. Sclereids are present in this zone. The epimatium is papery and fused with the exotesta surrounding the whole seed (Figure 2a,b). (ii). Exotesta: 10–14 layers of small and large rounded cells. (iii). Mesotesta: 10–16 layers of small and large cells. Several vascular traces, resin ducts and resin canals are present. (iv). Endotesta: 10–16 compact layers of small dense cells that are sclerified when the seed cone is mature. However, the cone is not woody. (v). Nucellus: 7–10 layers of large and small rounded cells that form the protective cover of the embryo. (vi). Gametophyte: 12–22 layers of large and small isodiametric cells with a straight embryo (about 0.6–0.8 × 0.2–0.3 mm in size). (vii). Receptacle: 10–15 layers of small and large rounded cells with four vascular traces in the centre of the receptaculum (Figure 3B).

#### 3.3.4. *Podocarpus spinulosus*

**Figure 4:**
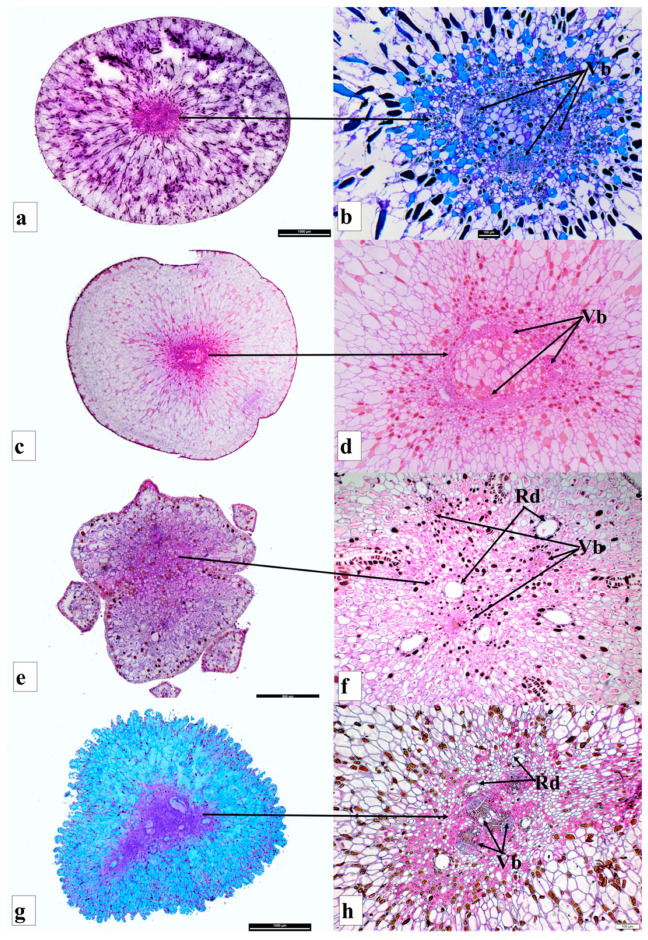
Receptacle anatomy of *Podocarpus oleifolius* cross section (**a**,**b**); *Podocarpus spinulosus* cross section (**c**,**d**); *Dacrydium cupressinum* cross section (**e**,**f**); and *Acmopyle pancheri* cross section (**g**,**h**). Shown are the vascular bundles (Vb) and resin canals (Rc).

*Podocarpus spinulosus* completes its reproductive cycle in one year and produces elongated oblong fleshy seed cones (11–22 × 6–7 mm). The inverted ovule is positioned on the blueish black fleshy receptacle. Six major zones can be observed in the cross section of the seed cone: (i). Epimatium:14–18 layers of small and large rounded and elongated cells. Sclereids are present in this zone. (ii). Exotesta: 12–16 layers of round and elongated cells. Several vascular traces, resin ducts, resin canals and sclereids are present in this zone. The epimatium is papery and fused with the exotesta surrounding the whole seed. (iii). Endotesta: 5–8 compact layers of small and large dense cells that become highly sclerified when the seed cone matures. However, the endotesta is not woody. (iv). Nucellus: 6–8 layers of dense cells form the protective cover of the embryo. (v). Gametophyte: 12–22 layers of large and small isodiametric cells with a straight embryo (about 0.6–0.9 × 0.2–0.4 mm in size). (vi). Receptacle: 9–18 layers of small and large rounded cells. The longitudinal section shows that three vascular bundles enter the receptaculum. The central vascular bundle enters the seed (Figure 4c,d).

#### 3.3.5. *Podocarpus elatus*

*Podocarpus elatus* completes its reproductive cycle in about one year, initiated from the last week of August in the new vegetative buds. It produces abundant elongated oblong fleshy seed cones, with one or sometimes two inverted ovules positioned on the bright red fleshy receptacle, which is formed by two bracts. Two weeks after seed cone initiation, a bract ovule complex appears. The peduncle extends the seed cone away from the shoot. This bract ovule complex differentiates into the nucellus and integument. Pollination and fertilization occur in November–December. Six major zones can be observed in the cross section of the seed cone: (i). Epimatium: 6–10 layers of small and large isodiametric cells. Sclereids are present in this zone. (ii). Exotesta: 18–26 layers of round and elongated cells. Several vascular traces, small and large resin canals (resin ducts at the end of the exotesta) and sclereids are present in this zone. The epimatium is papery and fused with the exotesta surrounding the whole seed (Figure 2g,q,h). (iii). Endotesta: 4–6 compact layers of small dense cells that become sclerified when the seed cone matures. However, the endotesta is not woody. (iv). Nucellus: 2–4 layers of small, dense cells form the protective cover of the embryo. (v). Gametophyte: 14–20 layers of large isodiametric cells with a straight embryo (about 0.7–1.2 × 0.3–0.5 mm in size). (vi). Receptacle: 14–18 layers of small and large rounded cells and six elongated vascular bundles arranged spirally, with several resin ducts.

### 3.4. Retrophyllum Seed Cone Morpho-Anatomy

*Retrophyllum comptonii* produces solitary reddish oblong-subglobose seed cones (10–20 × 7–15 mm). The seed cone is surrounded by 3–5 bracts. The ovule is inverted. Six major zones can be observed in the cross section of the seed cone: (i). Epimatium: 6–12 layers of large isodiametric cells. Sclereid cells are dominant in this zone. (iii). Exotesta: 16–20 layers of dense round cells. Several vascular traces, resin canals and sclereids are present in this zone. The epimatium is fleshy and fused with the exotesta surrounding the whole seed, forming a fleshy sarcotesta-like seed coat. (iii). Mesotesta: 6–8 layers of isodiametric cells, containing several vascular traces and resin ducts. (iv). Endotesta: 12–18 compact layers of small dense sclerified cells. This zone is hard and stony when the seed matures, forming a sclerotesta-like structure. (v). Nucellus: 4–8 layers of dense and smaller cells forming the protective cover of the embryo. (vi). Gametophyte: 8–12 layers of dense isodiametric cells with a straight embryo (about 0.4–0.9 × 0.2–0.4 mm in size).

### 3.5. Acmopyle Seed Cone Morpho-Anatomy

**Figure 5:**
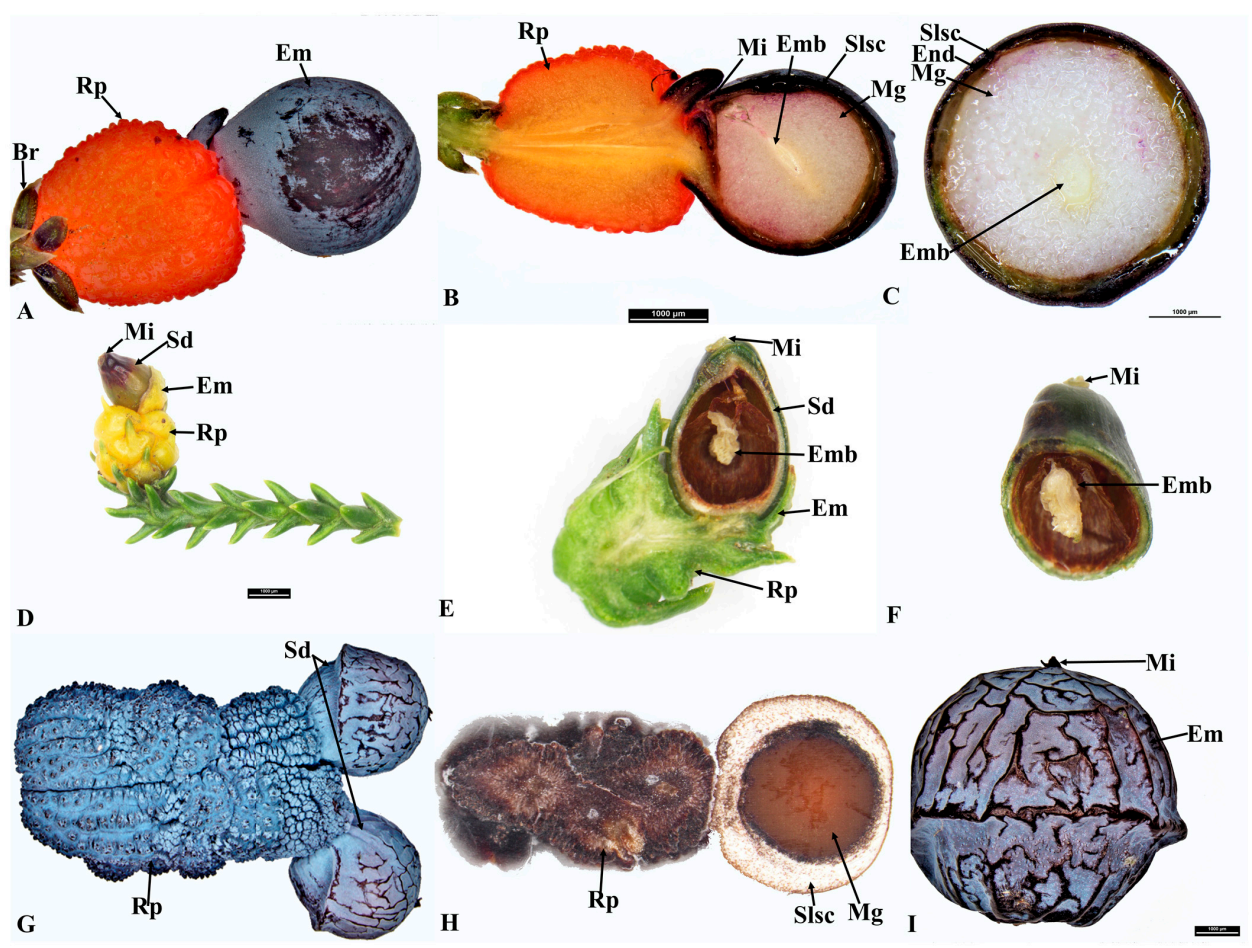
Seed cone of *Dacrycarpus dacrydioides* (**A**) longitudinal section (**B**) cross section (**C**); *Dacrydium cupressinum* (**D**), longitudinal section (**E**), cross section (**F**); and *Acmopyle pancheri* (**G**), longitudinal section (**H**), seed (**I**). Shown are the epimatium (Em), bract (Br), fleshy sarcotesta-like seed coat (Slsc), mesotesta (Mes), endotesta (End), megagametophyte (Mg), micropyle (Mi), embryo (Emb), resin canal (Rc), resin duct (Rd) and receptaculum (Rp).

*Acmopyle pancheri* seed cones initiate in September, pollination and fertilization occur in December and the cones mature in May–June. The elongated oblong (18–34 × 10–18 mm), reddish brown seed cones are produced on scale-leaved branches of the second order. The receptacle is verrucose, papillate and fleshy (Figure 5G). The peduncle and scaly leaves transform into a fleshy structure, forming the receptacle. The seed cone has 6–10 bracts, with one or sometimes two erect ovules. Seven major zones can be observed in the cross section of the seed cone: (i). Epimatium: 5–8 layers of small and large isodiametric cells, with sclereids dominant. (ii). Exotesta: 14–20 layers of dense round cells, including several vascular traces, resin canals and sclereids. The epimatium is papery and fused with the exotesta surrounding the whole seed. (iii). Mesotesta: 6–12 layers of small dense cells. (iv). Endotesta: 12–18 compact layers of small dense sclerified cells, forming a thick, woody and stony zone. (v). Nucellus: 4–8 layers of dense and smaller cells forming the protective cover of the embryo. (vi). Gametophyte: 8–12 layers of dense isodiametric cells with a straight embryo (about 0.3–0.6 × 0.1–0.2 mm in size). (vii). Receptacle: A fleshy elongate receptaculum is present, consisting of 12–18 layers of small and large rounded cells with four vascular bundles and resin ducts at the centre (Figure 4g,h).

### 3.6. Dacrycarpus Seed Cone Morpho-Anatomy

**Figure 6:**
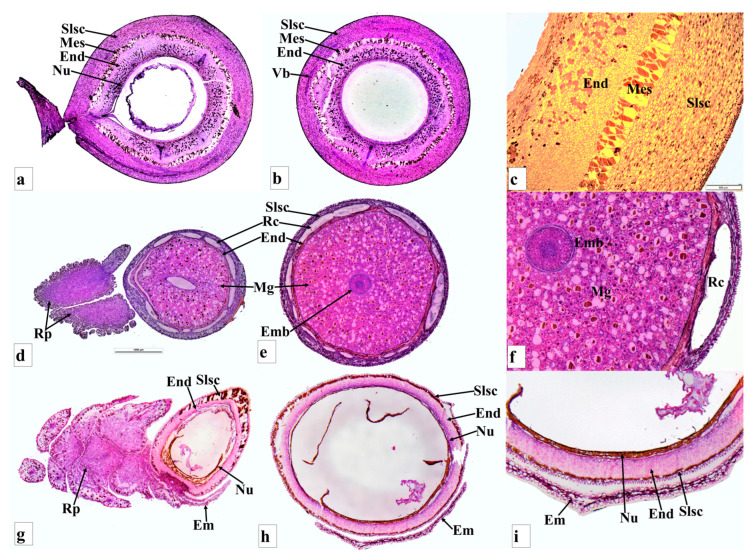
Anatomy of seed cones of *Afrocarpus falcatus* longitudinal section (**a**), cross section (**b**,**c**); *Dacrycarpus dacrydioides* longitudinal section (**d**), cross section (**e**,**f**); and *Dacrydium cupressinum* longitudinal section (**g**), cross section (**h**,**i**). Shown are the fleshy sarcotesta-like seed coat (Slsc), mesotesta (Mes), endotesta (End), epimatium (Em), megagametophyte (Mg), micropyle (Mi), embryo (Emb), nucellus (Nu), resin canal (Rc), resin duct (Rd) and receptaculum (Rp).

*Dacrycarpus dacrydioides* seed cones initiate in March and mature in January–February. The seed cones are elongated oblong (10–18 × 4–8 mm) and fleshy, with an orange –red colour. The peduncle and keel-shaped leaves transform into a fleshy receptacle. The seed cone usually has 2–3 bracts and one inverted ovule. Six major zones can be observed in the cross section of the seed cone: (i). Epimatium: 3–6 layers of elongated cells. (ii). Exotesta: 10–14 layers of dense small round and elongated cells, including several vascular traces and resin canals, which are elongated resin ducts. The epimatium is papery and fused with the exotesta surrounding the whole seed (Figure 6d–f). (iii). Endotesta: 4–6 compact layers of small dense sclerified cells. However, the endotesta is not woody. (iv). Nucellus: 3–6 layers of elongated irregular cells form the protective cover of the embryo. (v). Gametophyte: 10–15 layers of dense large isodiametric cells with a straight embryo (about 0.6–1.0 × 0.2–0.5 mm in size). (vi). Receptacle: A fleshy receptaculum is present, consisting of 12–18 layers of small and large rounded cells, with three vascular bundles and resin ducts. 

### 3.7. Dacrydium Seed Cone Morpho-Anatomy

*Dacrydium cupressinum* seed cones initiate in August–September and mature in March (~18 months later). The seed cones are elongated oblong (6–10 × 2–4 mm), fleshy and bright red. The fertile bracts transform into a swollen and fleshy bright-coloured structure at maturity. The seed cone has 8–14 bracts and one inverted ovule. An asymmetrical cup-like fleshy epimatium is also present (Figure 5D,E and Figure 6g–i). Six major zones can be observed in the cross section of the seed cone: (i). Epimatium: 3–8 layers of small irregular cells. The epimatium is a fleshy, asymmetrical, cup-like structure covering half of the seed. (ii). Exotesta: 1–2 layers of elongated cells. No vascular traces or resin canals are present. (iii). Endotesta: 8–18 compact layers of small dense sclerified cells. (iv). Nucellus: 6–10 layers of dense and smaller rounded cells, forming the protective cover of the embryo. (v). Gametophyte: 8–14 layers of dense isodiametric cells with a straight embryo (about 0.25–0.6 × 0.1–0.2 mm in size). (vi). Receptacle: 12–18 layers of small and large rounded cells, including three vascular bundles and several resin ducts (Figure 4e,f). 

### 3.8. Falcatifolium Seed Cone Morpho-Anatomy

*Falcatifolium papuanum* seed cones are solitary with a fleshy asymmetrical cup-like epimatium and receptaculum. The seed cones are obovoid and fleshy (10–16 × 4–6 mm). The peduncle and keel-shaped leaves transform into a fleshy red receptacle. The seed cone usually has 6–12 bracts. The ovule orientation is inverted, but this gradually changes to nearly erect when the seed cones are mature. Six major zones can be observed in a cross section of the seed cone: (i). Epimatium: 4–8 layers of small, elongated and irregular-shaped cells. (ii). Exotesta: 3–6 layers of small rounded and elongated cells, including vascular traces or resin canals. (iii). Endotesta: 6–14 compact layers of small dense sclerified cells. (iv). Nucellus: 4–8 layers of dense and small rounded cells forming the protective cover of the embryo. (v). Gametophyte: 8–16 layers of dense isodiametric cells with a straight embryo (about 0.3–0.6 × 0.15–0.25 mm in size). (vi). Receptacle: 10–18 layers of small and large isodiametric cells, with two vascular bundles and resin ducts. 

### 3.9. Comparison of Seed Cone Morpho-Anatomical Characters 

The seed cone shape of the species studied in both clades varies from obovoid (*Afrocarpus falcatus*, *Podocarpus henkelii*, *Retrophyllum comptonii*, *Dacrydium cupressinum* and *Falcatifolium papuanum*), to globose (*Nageia nagi*), to oblong (*N. wallichiana*, *Podocarpus elongatus*, *P. oleifolius*, *P. spinulosus*, *P. elatus*, *Dacrycarpus dacrydioides* and *Acmopyle pancheri*). The ovule orientation is inverted in all species except *A. pancheri*. The number of ovules per seed cone is usually one and sometimes two (Table 1). The testa is observed to be differentiated into three zones (exotesta, mesotesta and endotesta) or two zones (exotesta and endotesta). In the Podocarpoid clade, three zones are present in *Afrocarpus falcatus*, *Podocarpus henkelii*, *P. elongatus*, *P. oleifolius* and *Retrophyllum comptonii* and two zones in *Nageia nagi*, *N. wallichiana*, *Podocarpus spinulosus* and *P. elatus*. In the Dacrydioid clade, the testa is divided into two zones (exotesta and endotesta) in the species examined (*Dacrydium cupressinum*, *Dacrycarpus dacrydioides* and *Falcatifolium papuanum*). An epimatium is present in all species (Figure 1, Figure 2, Figure 3, Figure 4, Figure 5 and Figure 6). The epimatium and outer testa in all species, except *Dacrydium cupressinum* and *Falcatifolium papuanum*, are connate, forming a papery/coriaceous or fleshy sarcotesta-like seed coat. This sarcotesta-like seed coat is papery/coriaceous in all species studied except *Afrocarpus falcatus*, *Nageia nagi*, *Podocarpus henkelii* and *Retrophyllum comptonii*, which possess a fleshy sarcotesta-like seed coat, but lack a fleshy receptacle (Figure 1 and Figure 2). The mesotesta is woody and stony in *Acmopyle pancheri*, while the endotesta is woody and stony in *Afrocarpus falcatus*, *Podocarpus henkelii* and *Retrophyllum comptonii*. Species with a fleshy sarcotesta-like seed coat have a hard woody mesotesta or endotesta, except for *Acmopyle pancheri*, which has a papery sarcotesta-like seed coat at maturity. An asymmetrical cup-like fleshy epimatium is present in *Dacrydium cupressinum* and *Falcatifolium papuanum*. *Nageia wallichiana* and *N. motleyi* are the only species in the genus with a fleshy, bright red receptaculum. The receptacle surfaces of *Acmopyle pancheri*, *A. sahniana* and *Dacrycarpus dacrydioides* have a papillate appearance (Figure 3 and Figure 5). Whitish wax is present on both the receptacle and seed surface of most species, and in *Acmopyle pancheri* and *Afrocarpus falcatus*, the entire seed cones are covered by stomata containing white wax. Vascular bundles, resin canals and resin ducts are present in the testa of all species and are especially prominent in *Acmopyle sahniana*, *Dacrycarpus dacrydioides*, both *Nageia* species, *Retrophyllum comptonii*, all *Podocarpus* species examined and *Afrocarpus falcatus*. A system of several small vascular traces and resin ducts is present in the exotesta of *Afrocarpus falcatus*, *Podocarpus elongatus*, *P. henkelii*, *P. spinulosus*, *P. elatus*, *Nageia nagi*, *N. wallichiana* and *Retrophyllum comptonii*, and in the mesotesta of *Podocarpus oleifolius*. A similar complex system of vascular traces and resin canals is present in *Dacrycarpus dacrydioides* in the inner layers of the exotesta. A resinous layer or a system of enlarged resin ducts is present in the mesotesta of *Afrocarpus falcatus*, *Podocarpus elongatus*, *P. henkelii* and *Retrophyllum comptonii*; enlarged resin canals are present in the mesotesta of *Podocarpus henkelii* and the endotesta of *Podocarpus spinulosus* and *P. elatus*. A similar complex system of enlarged resin ducts is present in *Dacrycarpus dacrydioides* in the inner layers of the mesotesta. No such system was observed in *Dacrydium cupressinum* or *Falcatifolium papuanum*.

### 3.10. Seed Cone Morpho-Anatomical Traits and Structures

Both clades have evolved diverse structures and traits in their seed cones. 

#### 3.10.1. Fleshy Seed Cones 

The species of both clades produce fleshy seed cones, although the fleshy structures (receptaculum, epimatium or fleshy sarcotesta-like seed coat) differ among the genera. The ancestral state reconstruction of fleshiness and non-fleshiness in podocarps is not very meaningful in an evolutionary sense, as the fleshy structures differ across the clades, and sometimes within genera. All four genera of the Podocarpoid clade (*Afrocarpus*, *Podocarpus*, *Nageia* and *Retrophyllum*) and three genera of the Dacrydioid clade (*Dacrydium*, *Dacrycarpus* and *Falcatifolium*) produce fleshy seed cones, but the fleshy structures differ among the genera. A fleshy receptaculum and sarcotesta-like seed coat are the main fleshy structures.

#### 3.10.2. Epimatium Morphology

**Figure 7:**
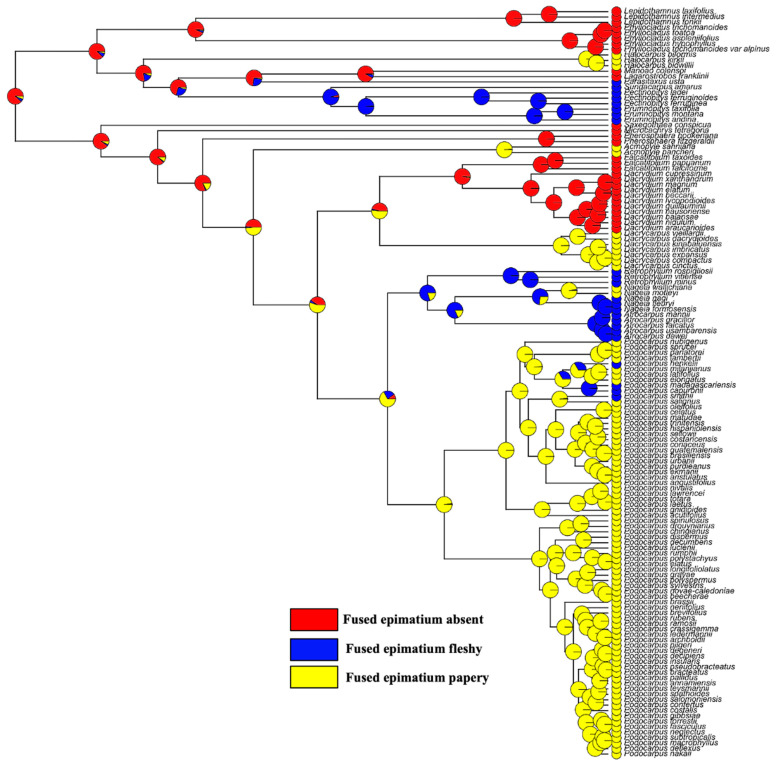
Character mapping of fused epimatium fleshiness and non-fleshiness in different genera of Podocarpaceae—maximum likelihood.

An epimatium is present in all species in both clades. The ancestral state reconstructions infer that the presence of an epimatium is an ancestral state in the Podocarpoid and Dacrydioid clades. However, the epimatium morphology differs among genera, from free to fused and from fleshy to papery. Ancestral state reconstructions indicate that the papery fused epimatium is an ancestral trait in the Podocarpoid clade and the fleshy fused epimatium evolved several times (Figure 7). An epimatium fused with the outer testa is present in all species of the Podocarpoid clade and evolved independently in *Dacrycarpus* (Dacrydioid clade). *Dacrydium* and *Falcatifolium* have a free asymmetrical cup-like epimatium that usually becomes fleshy when the seed cone is mature. *Dacrycarpus* and *Podocarpus* species with a fused epimatium have a papery morphology, except in *P. smithii*, *P. henkelii*, *P. madagascariensis* and *P*. *capuronii* and in four of the six species of *Nageia*, where the epimatium is fused to the outer testa and develops a fleshy sarcotesta-like seed coat, akin to that in *Afrocarpus* and *Retrophyllum* (Figure 1). 

#### 3.10.3. Receptaculum Morphology 

The presence of a fleshy receptacle is a characteristic feature in both clades. In most species of *Podocarpus*, the fleshy structure is a receptaculum (the exceptions are *P. smithii*, *P. henkelii*, *P. madagascariensis* and *P*. *capuronii*). A receptaculum is also present in two species of *Nageia* (*N*. *wallichiana* and *N*. *motleyi*) and all species of *Dacrydium*, *Dacrycarpus*, *Falcatifolium* and *Acmopyle* (Figure 4). The ancestral state reconstructions elucidate that the presence of a fleshy receptaculum is most likely an ancestral trait in both clades examined here (Supplementary Figure S2). The fleshy receptaculum is absent in some *Podocarpus* species (*P. smithii*, *P. henkelii*, *P. madagascariensis* and *P. capuronii*), which develop a fleshy fused epimatium with the outer testa, with a woody and stony endotesta. The receptaculum is usually brightly coloured. The seed cone bracts or scale leaves around the seed and the asymmetrical cup-like epimatium transform into a fleshy structure at seed cone maturity in *Dacrydium* and *Falcatifolium*. The receptacle in *Acmopyle* has a papillate surface (similar to *Dacrycarpus*) with white (waxy) stomata. 

#### 3.10.4. Testa Morpho-Anatomy

**Figure 8:**
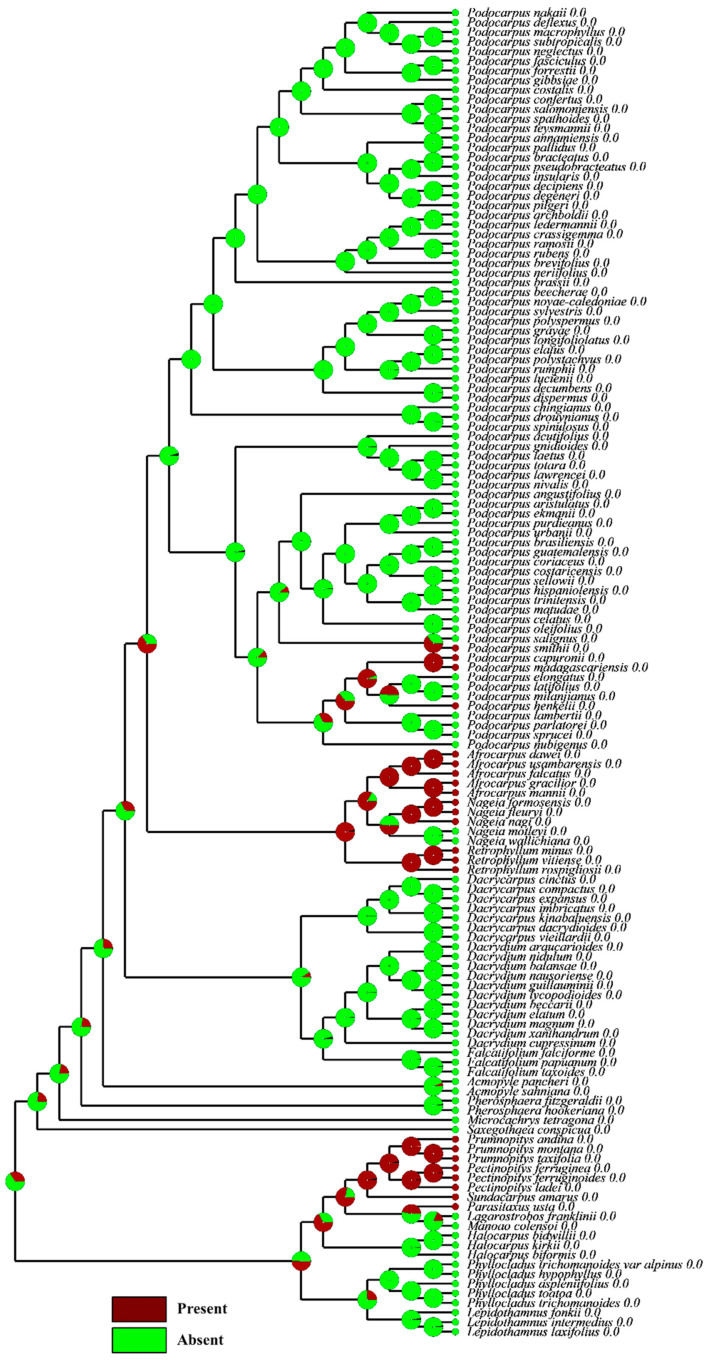
Character mapping of the fleshy sarcotesta-like structure in different genera of Podocarpaceae—maximum likelihood.

In *Dacrydium*, *Dacrycarpus*, *Falcatifolium*, *Nageia* (*N*. *wallichiana* and *N*. *motleyi*), *Podocarpus* (except *P. smithii*, *P. henkelii*, *P. madagascariensis* and *P*. *capuronii*) and *Acmopyle*, the testa is non-fleshy and has a papery structure. The endotesta is also non-woody in all these species, except *Acmopyle*, where the endotesta is woody and stony. The testa is fused with the epimatium, forming a fleshy sarcotesta-like seed coat, in *Retrophyllum*, *Afrocarpus*, *Nageia* and in some species of *Podocarpus* (e.g., *P. smithii*, *P. henkelii*, *P. madagascariensis* and *P*. *capuronii*). These species also have a hard woody mesotesta or endotesta. This strategy possibly evolved in these species to encourage endozoochory but to prevent digestion of the seed. The ancestral state reconstructions of the fleshy sarcotesta-like structure show that it evolved several times in the Podocarpoid clade (Figure 8). *Retrophyllum* has no fleshy receptaculum and is comparable to *Afrocarpus* in developing a fleshy sarcotesta-like seed coat. The main morpho-anatomical difference between these genera is the presence of a hard stony sclerotesta in *Afrocarpus* and *Retrophyllum* and a non-woody sclerotesta in most species of *Podocarpus* and two species of *Nageia*. Several vascular bundles and resin canals were present in the exotesta of all the *Podocarpus* species examined. 

#### 3.10.5. Ovule and Embryo Traits

Prior to pollination, ovules are inverted and the micropyle points towards the cone axis/base in all species of both clades. However, the micropyle of the mature seed cones of *Dacrydium*, *Falcatifolium* and *Acmopyle* points towards the apex, i.e., the ovule orientation changes post-pollination. The number of prosuspensor cells varies widely in the Podocarpaceae (Table 2), and it ranges from seven to twenty-three in the Podocarpoid clade and from five to fourteen in the Dacrydioid clade. The number is lower in other reported Podocarps (3–6), and there is variable reporting of the number of binucleate cells and elongating cells present. The reproductive cycle varies from one to two years. *Afrocarpus falcatus*, *Nageia* and *Dacrycarpus dacrydioides* complete their reproductive cycle in one year from seed cone initiation to the production of mature seed cones, while *Dacrydium cupressinum* and *Acmopyle pancheri* complete their reproductive cycle in two years. The reproductive cycle also varies among species of the same genus, e.g., *Podocarpus* species complete their reproductive cycle in one to two years, depending on the species.

## 4. Discussion 

### 4.1. The Dominance of Fleshy Seed Cones in Living Podocarps and Fossil Evidence

Most of the living podocarps (about 18 genera) produce fleshy seed cones. Fleshy seed cones are present in all genera of both clades. However, the fleshy structures are different (e.g., fleshy sarcotesta-like seed coat, bracts, receptacle and epimatium) and evolved multiple times. Podocarps, in general, evolved fleshy seed cones using different functional structures, e.g., receptaculum, epimatium, fleshy sarcotesta-like seed coat, aril or fleshy bracts [9,10,13,20]. 

Klaus and Matzke [6] reported that seven genera of the Podocarpaceae (including some *Podocarpus* species) have non-fleshy seed cones, while Herting et al. [8] reported that three genera of the Podocarpaceae (*Manoao*, *Saxegothaea* and *Pherosphaera*) have non-fleshy seed cones. Khan and Hill [9] suggest that 18 podocarp genera show fleshiness (the exceptions being *Saxegothaea* and *Pherosphaera*). This study confirms the presence of fleshy epimatium in *Dacrydium*, *Falcatifolium*, *Microcachrys* and *Lepidothamnus*, contrary to the study by Chen et al., [19]. However, the current study demonstrates that fleshy seed cones have evolved several times, using different functional structures. Klaus and Matzke [6] suggested that a reversion from fleshy to non-fleshy seed cones took place by the Early Eocene in the *Retrophyllum* + *Nageia* + *Afrocarpus* clade. The current study suggests no reversion to non-fleshy seed cones, because these genera have fleshy seed cones. Herting et al. [8] also considered the seed cones of *Retrophyllum* + *Nageia* + *Afrocarpus* clade as fleshy in their ancestral state reconstructions. Klaus and Matzke [6] considered a seed cone fleshy in the Podocarpoid clade if the taxa had a fleshy receptacle and were likely bird-dispersed. The fossil record demonstrates the presence of fleshiness or fleshy structures in the female cone of the Podocarpaceae by the Middle Cretaceous [32]. 

The fossil record also provides some evidence for the presence of some of these traits. Fossil seed cones of the extinct species *Podocarpus witherdenensis*, from the Eocene of Vegetable Creek, NSW, Australia, show the presence of a fleshy receptaculum and a non-fleshy fused epimatium [33]. The paleo data also suggest that the presence of fleshy cones, similar to crown podocarps, may have existed by the Early Cretaceous, in the Rajmahal flora in India [34]. Seed cones with a free epimatium that are regarded as belonging to an extinct species of the genus *Lepidothamnus* were recorded from the Middle Cretaceous of Winton, Queensland [32]. Similarly, some extinct podocarps (e.g., *Mataia*, *Nipaniostrobus*, *Harrisiocarpus gucikii* and *H. cracoviensis*) also show the presence of an epimatium-like structure [35,36,37,38,39]. The fossil cone records also show the presence of a fleshy receptaculum in *Dacrycarpus* in the Miocene–Eocene [33]. Seed cones with a fleshy receptaculum are recorded in fossil *Dacrycarpus guipingensis* from the Miocene of Guangxi, South China [40], *D. puertae* from the Eocene of Patagonia [41] and *D. mucronatus* from the Miocene of Balcombe Bay, Victoria, Australia [42].

### 4.2. Shifts in the Fleshy Structures 

In *Retrophyllum*, *Afrocarpus*, *Nageia* (except *N. wallichiana* and *N. motleyi*) and in some species of *Podocarpus* (e.g., *P. smithii*, *P. henkelii*, *P. madagascariensis* and *P. capuronii*), the seed coat (epimatium plus exotesta) is fleshy. The Podocarpoid clade exhibits a shift from a fleshy receptaculum to a fleshy sarcotesta-like seed coat on two separate occasions. A fleshy receptacle of several bright colours has been recorded in different species of *Podocarpus*, *Dacrydium*, *Dacrycarpus*, *Falcatifolium*, Acmopyle and two species of *Nageia*. The presence of a fleshy receptaculum is reconstructed as ancestral in both the Podocarpoid and Dacrydioid clades. However, in the Podocarpoid clade, the fleshy receptaculum is lost several times and reappears once in *Nageia*. This is also contrary to the study by Chen et al. [19], which suggested the absence of a fleshy receptacle. The only other genus with a fleshy receptaculum is *Lepidothamnus* [43]. The Podocarpoid clade shows a transition from a fleshy receptaculum to a fleshy fused epimatium and from a fleshy fused epimatium to a fleshy receptaculum. This transition is more evident in the Podocarpoid clade and was not limited to the ancestor of the *Afrocarpus*–*Nageia*–*Retrophyllum* clade but has also occurred within *Podocarpus* and *Nageia*. In the case of *Podocarpus*, this transition has occurred several times. A similar transition has occurred in the Prumnopityoid clade, suggesting that the evolutionary history of fleshiness in the Podocarpaceae is complex, because of the presence or absence of fleshiness and the functional structures that contribute to fleshiness among the seed cones (i.e., receptaculum, bracts and/or epimatium). The presence of a fleshy receptaculum in *Nageia* was previously reported by Mill, 2001 [44]. Klaus and Matzke [6] also reported that *Podocarpus latifolius*, *P. milanjianus* and *P. elongatus* have no fleshy receptacle, but our observations show that a fleshy receptaculum is present in *P. latifolius* and *P. elongatus*.

In contrast, the Dacrydioid clade is different because *Dacrycarpus* produces a fleshy receptaculum and a fused papery epimatium, while *Dacrydium* and *Falcatifolium* produce a free asymmetrical fleshy epimatium, that surrounds the seed and the subtending multiple bracts, or a fleshy receptaculum at maturity [45,46]. The epimatium is considered homologous to the ovuliferous scale of other conifers, and this conclusion is supported by Herting and Stützel, 2020 [47].

### 4.3. Significance of the Evolutionary Reconstruction of Fleshiness

Both clades produce brightly coloured fleshy seed cones. The ancestral reconstruction of the conifer seed cones implies that the ancestral seed cones of extant conifers were sclerified [8,45]. However, the fleshiness of the Podocarpaceae seed cones is an ancestral trait [8]. Klaus and Matzke [6] reported that fleshy seed cone structures appeared seven times in the Podocarpaceae independently and that the ancestral trait was non-fleshy. However, a reconstruction of fleshiness and non-fleshiness is not very meaningful in an evolutionary sense for podocarps, because the fleshy structures are quite different across clades, sometimes even within genera, and ancestral state reconstructions show that these fleshy structures have evolved multiple times in the Podocarpaceae.

### 4.4. Broader Perspective with other Podocarps

A broader podocarp perspective suggests that the type of morpho-anatomical adaptations and evolution of traits and structures seen in the Dacrydioid and Podocarpoid clades are typical of podocarps. A similar evolutionary pattern is present in the Prumnopityoid clade and Acmopyle [22]. Different functional structures of podocarps seed cones (sterile bracts, fertile bracts, epimatia and occasionally arils) produce fleshiness either singly or in combination. This shows that there is strong evolutionary pressure among the living podocarps, especially in the Podocarpoid clade, to produce fleshy seed cones. This supports the theory that species of the Dacrydioid and Podocarpoid clades evolved fleshy seed cones and broad flattened foliage as an adaptive strategy in the canopy-forming Cenozoic Forest evolution [2,48]. Fleshy fruits in podocarps are generally associated today with tropical forests [49]. A parallel shift in the seed cone size is also noticeable with Cenozoic Forest evolution [50]. The Podocarpoid clade species have relatively large seed cones (Supplementary Figure S4). Furthermore, Leslie et al. [50] reported that seed sizes are generally larger in animal dispersed species [5]. The number of seeds per cone varies in the Podocarpoid clade from one to two, which have, for example, been reported in *Podocarpus* [51]. Most genera in the Prumnopityoid clade (except Parasitaxus) possess one to two or else multiple seeds per cone, as does Acmopyle and the relict genera *Microcachrys*; Saxegothaea and Pherosphaera have multiovulate (seeded) cones [52]. Species with one or two seeds per cone generally have larger seed cones than the multiovulate cone. All species of both the Dacrydioid and Podocarpoid clades have inverted ovules. The Prumnopityoid clade also has inverted ovules. Pherosphaera, Phyllocladus and Acmopyle are the only three genera with mature erect ovules. In Acmopyle, ovules are usually horizontally inclined to the fertile bract initially and become erect after pollination [12].

### 4.5. Evolution of Morphological Structures and Animal Dispersal

The Dacrydioid and Podocarpoid clades show a remarkable tendency towards the evolution of fleshiness through a complex mechanism. Fleshiness has probably evolved through several different ways, in order to attract animals to disperse the seeds. Around 40% of living conifer species show fleshy morphologies [22]. Eighteen podocarp genera show fleshy structures that support the zoochorous dispersal of seed cones. The seed cone of podocarps is generally dispersed by birds and small animals, which are attracted by the brightly coloured fleshy structures [9,10,13]. Eleven podocarp genera show features consistent with avian endozoochory [6,53]. For example, masting is well known in many temperate podocarps, which may favour zoochory [54]. Both clades considered here show zoochory as the main seed dispersal strategy. For example, in the Podocarpoid clade, *Podocarpus* is the most dominant and widely distributed genus in Podocarpaceae [13]. Most *Podocarpus* species produce seed cones with a colourful, fleshy receptacle and are mainly dispersed through endozoochory, with frugivory being dominant (Table 1). *Afrocarpus falcatus* is reported to be dispersed by birds and several other vertebrates, (Table 1) because its fleshy epimatium and testa complex serve as a food source [43,55]. *Retrophyllum, Nageia* and *Acmopyle* produce colourful fleshy seed cones that also support zoochory [45,52,56]. Klaus and Matzke [6] reported that the morphological characteristics of the *Retrophyllum* seed cone do not support avian endozoochory, although they regarded the presence of a fleshy receptaculum as the key evidence. *Retrophyllum minus* is a rheophytic species at low elevations in New Caledonia, for which hydrochory has been reported [56,57]. The seed cone morphology of the Dacrydioid clade also supports zoochory, as all species produce fleshy seed cones (Table 1). The fleshy receptaculum of *Dacrycarpus dacrydioides* helps in the bird dispersal of seed cones [58,59]. The fossil record shows the unique geographic distribution of *Dacrycarpus* and is a good example of probable long-distance dispersal [40,41]. The fossil seed cone morphology also supports zoochory and bird dispersal. For example, the fossil seed cone records from Patagonia (Eocene) and South China (Miocene) show the presence of a fleshy receptacle [40,41]. The seed cones of *Dacrydium cupressinum* are dispersed by frugivory (Table 1). These cones are small and favour local dispersal (10–40 m) by wind [60], but they are well adapted for frugivory when the fleshy receptacle is fully developed [61,62,63,64,65,66]. *Falcatifolium* is also reported to be dispersed through zoochory (Table 1). The living species of the Dacrydioid and Podocarpoid clades have evolved fleshy and coloured seed cone structures and demonstrate a shift in the fleshy parts, and adaptation to endozoochory as the main seed dispersal strategy [6].

## 5. Conclusions

The Podocarpoid and Dacrydioid clades produce fleshy seed cones and have zoochory as their main dispersal strategy. Ancestral state reconstruction shows that although fleshiness in both clades is an ancestral trait, this fleshiness is achieved through diverse and complex mechanisms of similar and different functional structures, e.g., the receptaculum, epimatium and fleshy sarcotesta-like seed coat (fleshy fused epimatium with testa). The Podocarpoid clade follows a similar evolutionary pathway in achieving fleshiness (e.g., a shift in fleshiness from the receptaculum to the epimatium fused with the testa and vice versa). Similarly, the Dacrydioid clade shows both receptaculate seed cones with a papery, fused epimatium and receptaculate seed cones with a fleshy, free epimatium. This shows that in podocarps, fleshy seed cones have evolved via different functional structures, suggesting that the evolutionary pressure to evolve fleshy cones is high in these podocarps and they have the genetic and morphological flexibility to respond to this pressure in several different ways. This provides an exciting opportunity for investigating the evolutionary history of fleshy cones in conifer and connections between plants and animals in the early Cenozoic.

## Figures and Tables

**Table 1:** Seed cone morphological and anatomical, and qualitative and quantitative characteristics for the Podocarpoid and Dacrydioid clades.

Characters	*Afrocarpus falcatus*	*Nageia wallichiana*	*Nageia nagi*	*Podocarpus henkelii* (subgenus *Podocarpus*)	*Podocarpus elongatus* (subgenus *Podocarpus*)	*Podocarpus oleifolius* (subgenus *Podocarpus*)	*Podocarpus spinulosus* (subgenus *Foliolatus*)	*Podocarpus elatus* (subgenus *Foliolatus*)	*Retrophyllum comptonii*	*Dacrydium cupressinum*	*Dacrycarpus dacrydioides*	*Falcatifolium papuanum*	*Acmopyle pancheri*
Reproductive cycle	1 year	1 year	1 year	1 year	1 year	1 year	1 year	1 year	1 year	2 years	1 year	1 year	1–2 years
Cone shape	obovoid	ovoid	globose	obovoid	ovoid	ovoid	ovoid	ovoid	ovoid-subglobose	obovoid	ovoid	obovoid	ovoid
Cone size (mm)	12–20 × 8–16	18–38 × 6–10	12–18 × 10–16	15–20 × 8–12	13–20 × 4–8	12–22 × 4–7	11–24 × 6–7	25–45 × 10–20	10–20 × 7–15	6–10 × 2–4	10–18 × 4–8	10–16 × 4–6	18–34 × 10–18
Colour	yellowish	reddish	brown	yellowish	reddish	reddish	dark purple	dark purple	reddish	reddish	reddish	reddish	reddish-brown
Number of seeds per cone	1	1–2	1	1	1	1–2	1	1–2	1–2	1	1–2	1	1–2
Seed size (mm)	8–18 × 6–14	14–20 × 10–16	10–15 × 8–12	12–17 × 4–6	6–12 × 3–5	5–8 × 3–6	8–10 × 5–7	15–20 × 12–15	7–17 × 5–12	2.5–6 × 2–3	6–10 × 4–8	6–7 × 4–5	6–8 × 5–7
Seed surface	rugose	rugose	rugose	rugose	smooth	smooth	rugose	rugose	rugose	smooth	smooth	smooth	rugose
Seed colour	brown	brown	brown	light brown	brown	brown	brown	purplish black	brown	brownish black	purplish black	dark brown	brown
Ovule orientation	inverted	inverted	inverted	inverted	inverted	inverted	inverted	inverted	inverted	inverted	inverted	inverted	erect
Cuticle	thick	thick	thick	thick	thin	thin	thin	thin	thick	thick	thin	thin	thick
Epidermal layers	1	1–2	1–2	1	1	1	1–2	1	1	1	1	1	1
Epidermal cell shape	rectangular	round–triangular	round–triangular	round–triangular	round–triangular	rectangular	round–triangular	round–triangular	isodiametric	dome-shaped	dome-shaped	dome-shaped	triangular
Exotesta	fleshy	coriaceous	coriaceous	fleshy	coriaceous	coriaceous	coriaceous	coriaceous	fleshy	coriaceous	coriaceous	coriaceous	coriaceous
Exotesta layers of cells	10–18	14–22	12–20	12–18	8–13	10–14	12–16	18–26	16–20	1–2	10–14	3–6	14–20
Mesotesta layers of cells	3–5	–	–	16–22	3–5	10–16	–	–	6–8	–	–	–	6–12
Endotesta layers of cells	20–32	12–20	10–18	12–20	6–12	10–16	5–8	4–6	12–18	8–10	4–6	6–14	12–18
Nucellus layers	4–10	5–10	6–12	6–12	8–14	7–10	6–8	2–4	4–8	6–10	3–6	4–8	4–8
Embryo (megagametophyte) shape	straight	straight	straight	straight	straight	straight	straight	straight	straight	straight	straight	straight	straight
Embryo size (mm)	0.8–1.2 × 0.3–0.5	0.8–1.7 × 0.3–0.5	0.8–1.5 × 0.3–0.4	0.65–1.2 × 0.2–0.4	0.5–0.9 × 0.2–0.3	0.6–0.8 × 0.2–0.3	0.5–1.3 × 0.3–0.5	0.7–1.2 × 0.3–0.5	0.4–0.9 × 0.2–0.4	0.25–0.6 × 0.1–0.2	0.6–1.0 × 0.2–0.5	0.3–0.6 × 0.15–0.25	0.3–6 × 0.1–0.2
Bracts	1–2	4–7	4–6	1–2	2	2	2–4	2	3–5	8–14	2–3	6–12	4–10
Stomata on bracts	present	present	present	present	present	present	present	present	present	present	present	present	present
Epimatium	present	present	present	present	present	present	present	present	present	present	present	present	present
Epimatium shape	fleshy and fused with outer testa surrounding the whole seed	papery and fused with outer testa surrounding the whole seed	fleshy and fused with outer testa surrounding the whole seed	fleshy and fused with outer testa surrounding the whole seed	papery and fused with outer testa surrounding the whole seed	papery and fused with outer testa surrounding the whole seed	papery and fused with outer testa surrounding the whole seed	papery and fused with outer testa surrounding the whole seed	fleshy and fused with outer testa surrounding the whole seed	fleshy asymmetrical cup-like	papery and fused with outer testa surrounding the whole seed	fleshy asymmetrical cup-like	papery and fused with outer testa surrounding the whole seed
Epimatium structure	fleshy	fleshy	fleshy	fleshy	papery	papery	papery	papery	fleshy	fleshy	papery	fleshy	papery
Epimatium colour	yellowish	purple	purple	yellowish	purple	olive green	dark purple	purplish black	reddish	brownish black	purplish black	reddish brown	brown
Receptaculum	absent	present	absent	absent	present	present	present	present	absent	present	present	present	present
Receptaculum colour	-	reddish	-	absent	bright red	yellowish red	blueish black	blueish black	-	reddish	orange–red	reddish	reddish brown
Sclereids	present	present	present	present	present	present	present	present	present	present	present	present	present
Resin canals	present	present	present	present	present	present	present	present	present	present	present	present	present
Dispersal	zoochory [birds, bats (*Rousettus aegyptiacus*), monkeys (*Chlorocebus pygerythrus),* woodland dormouse (*Graphiurus murinus*) and Verreaux’s mouse (*Myomyscus verreauxii*)]	zoochory	zoochory	zoochory (birds)	zoochory (birds)	zoochory (birds)	zoochory (birds)	zoochory (birds)	zoochory	zoochory [New Zealand bellbird (*Anthornis melanura*) and Tūī (*Prosthemadera novaeseelandiae*), Kākāpō (*Strigops habroptilus*)]	zoochory [New Zealand pigeon (*Hemiphaga novaeseelandiae*), Tūī (*Prosthemadera novaeseelandiae*) and New Zealand bellbird (*Anthornis melanura*), *Anthornis melanura*, *Hemiphaga novaeseelandiae*, *Prosthemadera novaeseelandiae*]	zoochory (birds)	zoochory (birds)

**Table 2:** Number of prosuspensor and binucleate embryonic cells in Podocarpaceae.

Taxon	Number of Prosuspensor Cells	Number of Binucleate Embryonic Cells	Reference
Podocarpoid Clade			
*Afrocarpus*	18–23	9–11	Buchholz, 1941 [30]
*Nageia*	18–23	7, 9–11	#
*Podocarpus glomeratus*	11–13	2–3	#
*P. chinensis*	14–15	1–2	#
*P. lawrencei*	7–10	1–2	#
*P. nivalis*	7–10	1–2	#
*P. totara*	7–10	1–2	#
*P. laetus*	7–10	1–2	#
*P. urbanii*	11–13	2–3	#
Dacrydioid Clade			
*Dacrycarpus dacrydioides*	11–14	4 or 5	Buchholz, 1941 [30]
*Dacrydium*	7–11	5–9	#
Other Podocarpaceae			
*Phyllocladus*	4–6	9–12	Doyle and Looby, 1939 [31]
*Saxegothaea*	3–4	10–12	#
*Pectinopitys ferruginea*	7–9	7–9	Buchholz, 1941 [30]
*Prumnopitys taxifolia*	6–9	9–12	#

## Data Availability

Data are contained within the article and supplementary materials. And supplementary data available at https://doi.org/10.25909/17125403.

## Supplementary Materials

The following supporting information can be downloaded at: https://www.mdpi.com/article/10.3390/plants12223903/s1, Figure S1. Longitudinal (A) and cross section (B) of *Nageia nagi*; Figure S2. Character mapping of the receptaculum presence in different genera of Podocarpaceae using RASP 4.2 (Reconstruct Ancestral State) Maximum likelihood; Figure S3. Seed cone (A) and cross section (B) of *Podocarpus lawrencei*; Figure S4. Variation in seed cone size; L (length) and W (width). Species of Podocarpoid clade shows larger seed cone size as compared to Dacrydioid clade.

## Abbreviations

Rp = receptaculum/podocarpium, Ar = aril, Em = epimatium, Emr = epimatium ridge, Fs = fertile scale, Fsbr = fleshy sterile bracts, Ax = cone axis, Mi = micropyle, Ov = ovule, Ep = epidermis, Nu = nucellus, Ts = testa, Exo = exotesta, Mes = mesotesta, End = endotesta, Mg = megagametophyte, Emb = embryo, Mi = micropyle, Br = bract, Sd = seed, Vb = vascular bundle, Rc = resin canal, Rd = resin duct, Sc = seed cone, Sl = sclereid, Slsc = sarcotesta-like seed coat, Nc = nucellar cap, In = integument, Pd = peduncle, Cu = cuticle, Rl = resin layer, Sls = sclerotesta-like structure.
