# Supplementary material for: Detailed Seed Cone Morpho-Anatomy Provides New Insights into Seed Cone Origin and Evolution of Podocarpaceae; Podocarpoid and Dacrydioid Clades
Source: Plants (Basel). 2023 Nov 19;12(22):3903. doi: 10.3390/plants12223903 (PMC10674377; doi:10.3390/plants12223903)
Supplement: Supplementary file 1 [file plants-12-03903-s001.zip › plants-2558179-supplementary.pdf]

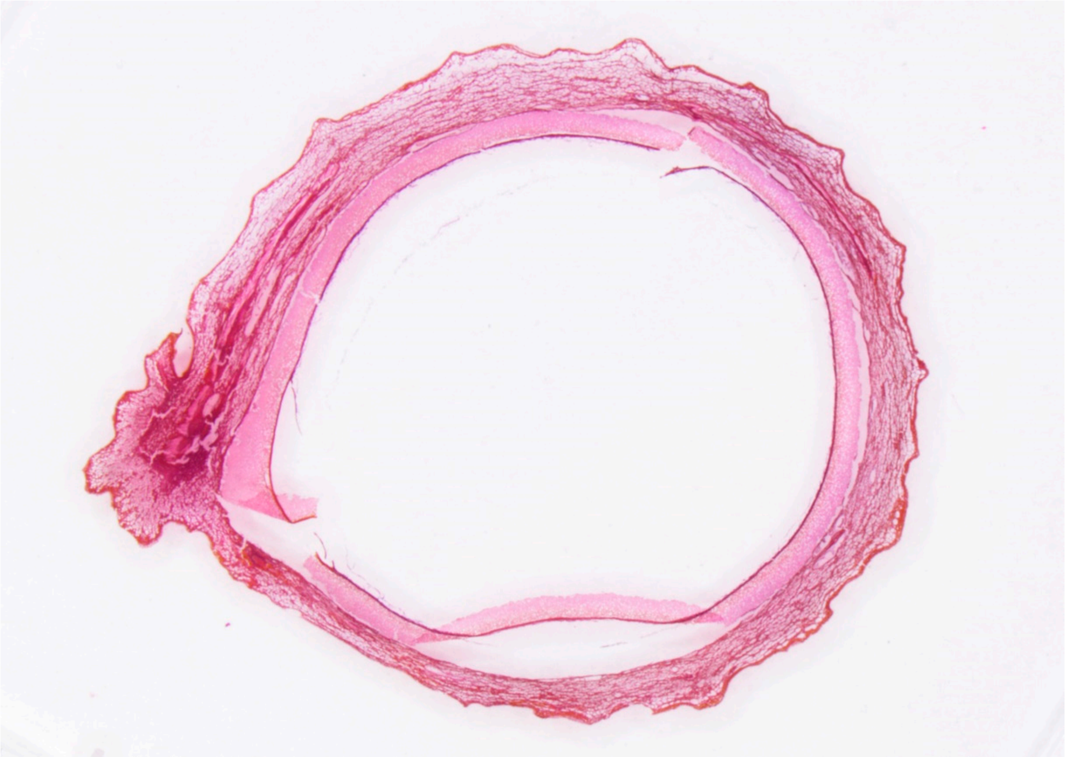

A)

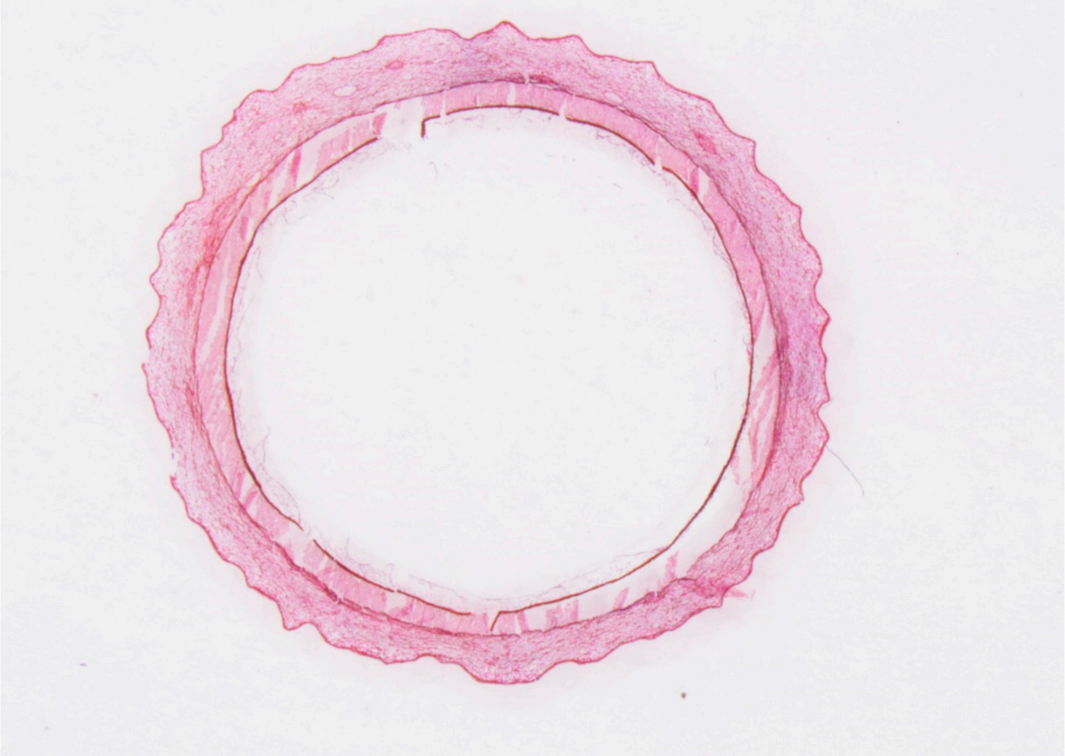

(B)

**Figure S1**. Longitudinal (**A**) and cross section (**B**) of *Nageia nagi*.

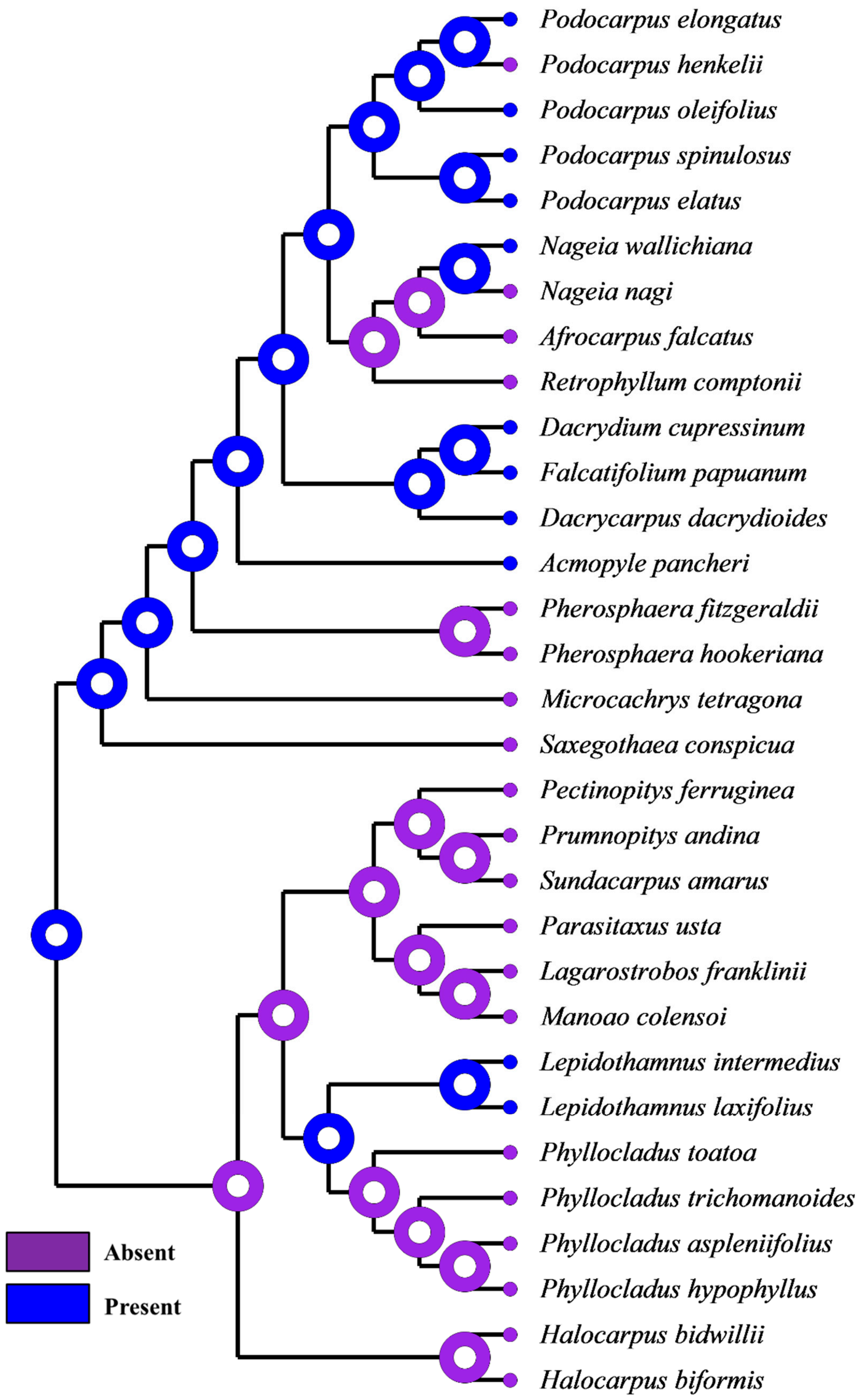


**Figure S2.** Character mapping of the receptaculum presence in different genera of Podocarpaceae using RASP 4.2 (Reconstruct Ancestral State) Maximum likelihood.

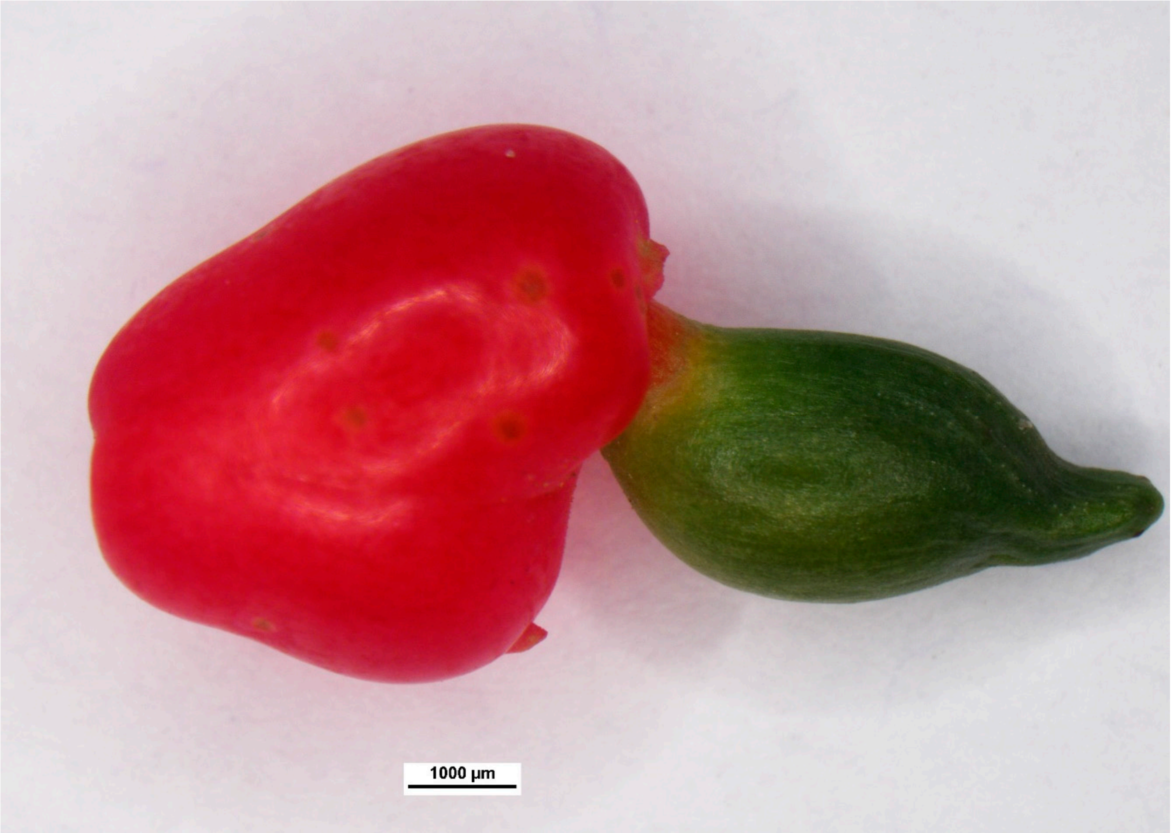


A)

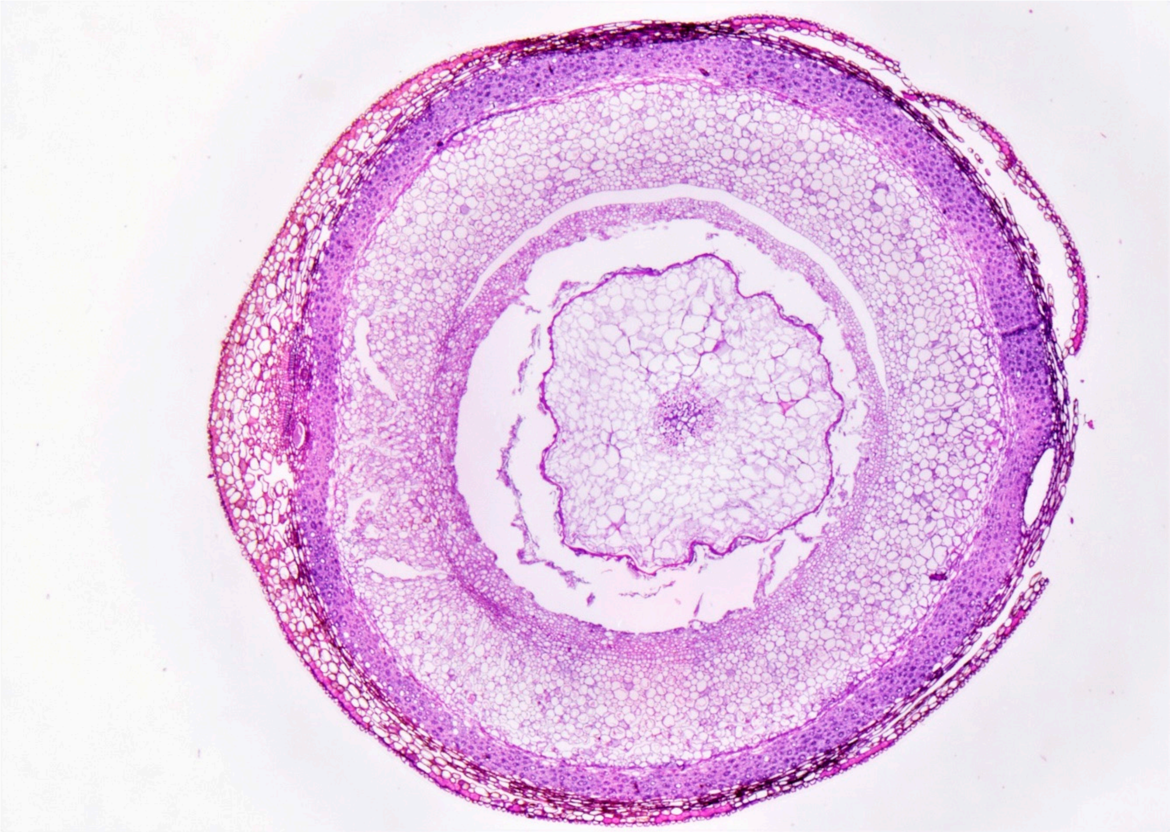

B)

**Figure S3.** Seed cone (**A**) and cross section (**B**) of *Podocarpus lawrencei*.

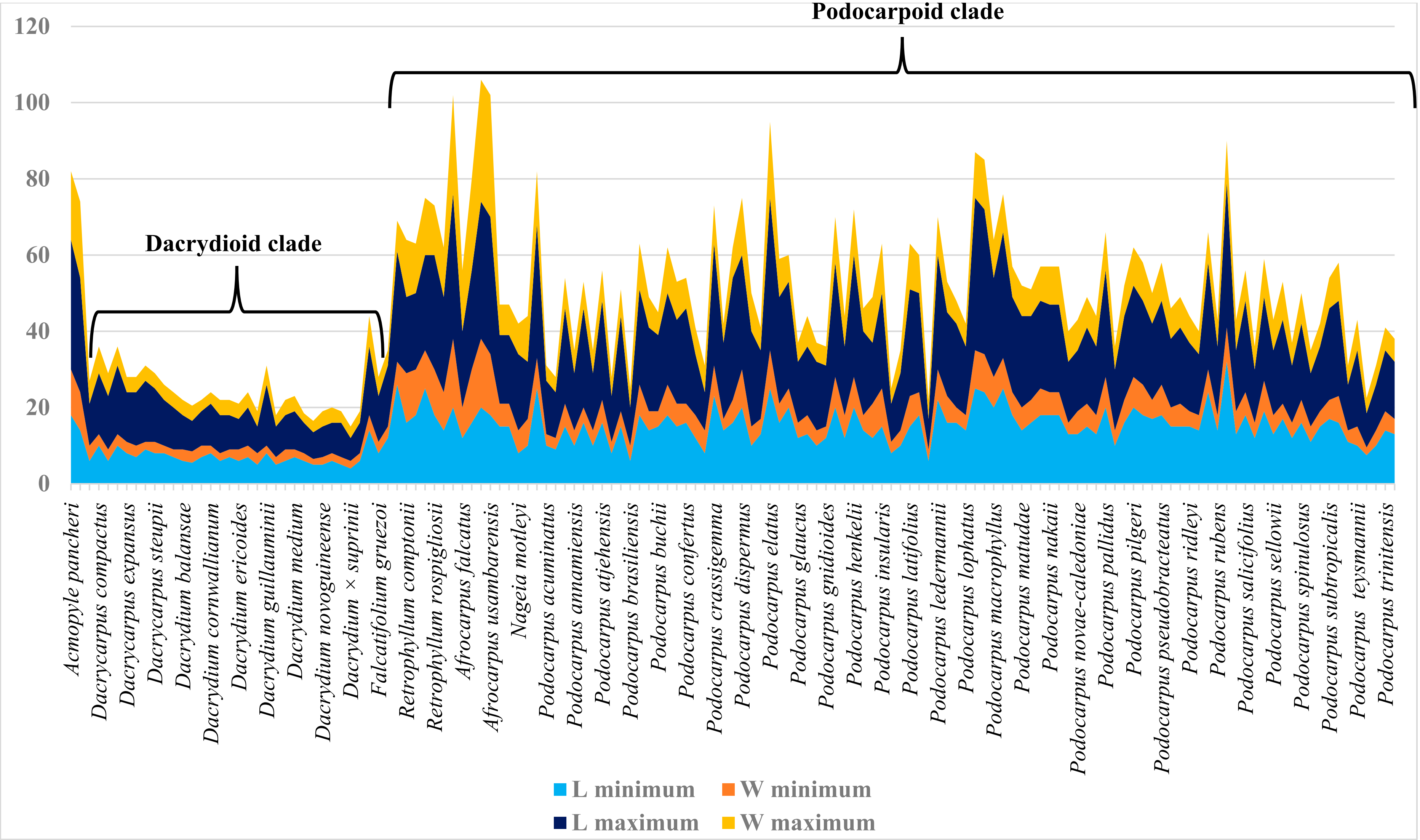


**Figure S4.** Variation in seed cone size; L (length) and W (width). Species of Podocarpoid clade shows larger seed cone size as compared to Dacrydioid clade.
